# An ounce of “superscreener”: A novel cancer screening program targeting unattached individuals

**DOI:** 10.17269/s41997-026-01169-y

**Published:** 2026-03-19

**Authors:** Anna N. Wilkinson, Kate Volpini, Amriya Naufer, Andrea Miville, Chantal Lalonde, Erika Kamikazi, Sarah Hepworth-Junkin

**Affiliations:** 1 Department of Family Medicine, University of Ottawa, Ottawa, ON Canada; 2 Champlain Regional Cancer Program at The Ottawa Hospital, Ottawa, ON Canada; 3 Chronic Disease Prevention & Wellbeing, Injury Prevention and Health Equity Programs at the Eastern Ontario Health Unit, Cornwall, ON Canada; 4 Community Engagement Team, Health Equity, Diversity, and Inclusion Program, Ottawa Public Health, Ottawa, ON Canada

**Keywords:** Cancer screening, Unattached individuals, Neverscreener, Primary care crisis, Dépistage du cancer, Personne sans attaches, Personne jamais dépistée, Crise des soins primaires

## Abstract

**Setting:**

Cancer screening enables early detection, reducing morbidity, mortality, and healthcare costs. Canada’s primary care crisis has left many individuals unattached, creating barriers to cancer screening and exacerbating disparities.

**Intervention:**

The Champlain Screening Outreach (CSO) program provided cancer screening to unattached individuals and underserved populations. CSO collaborated with public health, community health centers, and community organizations to increase engagement with typically underscreened populations. A nurse practitioner superscreener conducted screening consultations, ordered tests, and followed up on results. Data from the program’s inaugural year were analyzed, including participation, test completion, high-risk referrals, and abnormal findings. Sociodemographic data characterized unattached and neverscreener populations.

**Outcomes:**

In its first year, CSO conducted 527 consultations and facilitated 786 screening tests. High-risk referrals were significantly higher than provincial rates for colorectal (41.5% vs. 2.6%, *p* < 0.01) and breast cancer (27.3% vs. 1.1%, *p* < 0.01). Among participants, 195 (36.4%) were neverscreeners. Abnormal result rates were elevated across cancer types: cervical (5%), breast (13.1%), colon (28.1%), and lung (16.6%). FIT return rates (53.3%) were lower than the provincial average. CSO participants predominantly resided in lower-income neighborhoods with high immigrant and visible minority populations. Neverscreeners were more likely to experience neighborhood instability.

**Implications:**

CSO effectively improved cancer screening access for unattached individuals and reduced screening disparities. Elevated rates of abnormal findings and high-risk referrals highlighted the importance of collaboration with community organizations to reach underscreened populations and the efficacy of targeted screening assessments. CSO’s outcomes demonstrate its potential as a centralized, scalable, and proactive solution to enhance cancer screening equity and mitigate the impact of the primary care crisis.

## Résumé

**Contexte:**

Le dépistage du cancer permet une détection précoce, réduisant ainsi la morbidité, la mortalité et les coûts des soins de santé. La crise des soins primaires au Canada a laissé de nombreuses personnes sans attaches avec le système de santé, créant des obstacles au dépistage du cancer et exacerbant les disparités.

**Intervention:**

Le Programme d’accès au dépistage Champlain a permis de proposer un dépistage du cancer aux personnes sans attaches avec le système de santé et aux populations défavorisées. Le Programme a collaboré avec les services de santé publique, les centres de santé communautaires et les organismes communautaires afin d’accroître l’engagement des populations généralement sous-dépistées. Une infirmière praticienne « super dépisteuse» a mené des consultations de dépistage, prescrit des tests et assuré le suivi des résultats. Les données de la première année du programme ont été analysées, notamment la participation, la réalisation des tests, les orientations vers des spécialistes pour les cas à haut risque et les résultats anormaux. Les données sociodémographiques ont permis de caractériser les populations isolées et celles qui n’avaient jamais subi de dépistage.

**Résultats:**

Au cours de sa première année, le Programme d’accès au dépistage Champlain a mené 527 consultations et facilité 786 tests de dépistage. Les recommandations vers des spécialistes pour des cas à haut risque étaient nettement plus élevées que les taux provinciaux pour le cancer colorectal (41,5% contre 2,6%, *p* < 0,01) et le cancer du sein (27,3% contre 1,1%, *p* < 0,01). Parmi les participants, 195 (36,4%) n’avaient jamais subi de dépistage. Les taux de résultats anormaux étaient élevés pour tous les types de cancers: cancer du col de l’utérus (5%), cancer du sein (13,1%), cancer du côlon (28,1%) et cancer du poumon (16,6%). Les taux de retour des tests FIT (53,3%) étaient inférieurs à la moyenne provinciale. Les participants au Programme résidaient principalement dans des quartiers à faibles revenus, avec une forte population d’immigrants et de minorités visibles. Les personnes n’ayant jamais subi de dépistage étaient plus susceptibles de vivre dans des quartiers instables.

**Implications:**

Le Programme d’accès au dépistage Champlain a efficacement amélioré l’accès au dépistage du cancer pour les personnes sans attaches et réduit les disparités en matière de dépistage. Les taux élevés de résultats anormaux et de recommandations vers des soins à haut risque ont mis en évidence l’importance de la collaboration avec les organismes communautaires pour atteindre les populations sous-dépistées et l’efficacité des évaluations de dépistage ciblées. Les résultats du Programme démontrent son potentiel en tant que solution centralisée, évolutive et proactive pour améliorer l’équité en matière de dépistage du cancer et atténuer l’impact de la crise des soins primaires.

## Introduction

Screening for colorectal, breast, lung, and cervical cancers facilitates early detection, reducing morbidity, mortality, and healthcare costs (Goddard et al., 2024; Wilkinson et al., 2025). In Canada, guidelines exist for average risk cancer screening, and several provinces have specific high-risk screening programs (Canadian Task Force on Preventive Health, 2024; Cancer Care Ontario, 2025). Cancer screening uptake is suboptimal, with over one-third of eligible individuals overdue for colorectal cancer (CRC), and one-quarter missing breast and cervical cancer screening (Abel-Rahman, 2021; Lofters et al., 2019). Inequities are evident in cancer screening participation, with decreased screening rates noted in individuals of lower socioeconomic status and in immigrants (Blair et al., 2019; Elit et al., 2012; Khadikar et al., 2013; Lofters et al., 2019).

In Canada, primary care providers (PCPs) counsel, order, and follow up on cancer screening tests. However, 6.5 million people, or one in five, Canadians are “unattached” or do not have a regular PCP (Duong & Vogel, 2023). In Ontario, 25% of the population is predicted to be unattached by 2026 (Ontario College of Family Physicians, 2023). Unattached individuals are at risk of missing essential cancer screenings.

The Champlain Screening Outreach (CSO) program, created in response to the primary care crisis, represents a novel, proactive approach to cancer screening (The Ottawa Hospital, 2023; Grant, 2023). CSO aims to improve access to cancer screening while addressing participation disparities. The program utilizes a nurse practitioner (NP) “superscreener” with a threefold mandate: (1) cancer screening education; (2) screening for unattached individuals; and (3) partnership with community organizations to provide outreach cancer screening to underserved populations. This study evaluates the efficacy and equity of the CSO program in its first year, specifically assessing screening volumes and patterns, abnormal rates, and positive predictive values of screens relative to regional and provincial data.

## Methods

### Program description

The CSO program was established and funded by the Champlain Regional Cancer Program, beginning in July 2023. The program consisted of a part-time NP superscreener with no administrative support who provided cancer screening for the Champlain region of Ontario, an area including Ottawa and surrounding counties with a population of approximately 1.3 million. Eligible unattached individuals in the Champlain region self-referred to CSO via telephone or online booking. Participant recruitment strategies included social media and media, as well as posters in high-traffic areas for unattached individuals, such as emergency departments. The success of CSO was in large part driven by engagement and collaboration with regional partners, including public health units, Ontario health teams, the city of Ottawa, and various community organizations. Notably, collaboration with regional public health units played a crucial role in disseminating information, identifying eligible participants, and facilitating educational outreach. The Ottawa Public Health Community Engagement Team leveraged existing partnerships with community organizations, community groups, and communication channels established during the COVID-19 pandemic to identify underserved populations and support their engagement with CSO. This included translating educational materials into French, Arabic, Somali, and simplified Chinese, distributing resources, identifying outreach opportunities, and connecting CSO with local community organizations. Additionally, the Eastern Ontario Health Unit conducted public press releases, awareness campaigns, and conducted strategic partner outreach activities to promote cancer screening and the CSO program. Both health units provided clinical space for local screening appointments, reducing access barriers for community members. As well, the Eastern Ontario Health Unit facilitated the delivery of cervical screening samples to the laboratory, maximizing the NP Superscreener’s capacity during pap clinic days. Health units engaged in staff education to ensure staff were aware of CSO cancer screening services and could provide interested participants with the program contact information or assist with hands-on online booking support.

Individuals qualified for the CSO program if they were (1) living in the Champlain region; (2) eligible for screening; (3) not symptomatic; (4) covered by OHIP or IFHP; and (5) not attached to a PCP. Participants could self-schedule online or by telephone to book a half hour telephone screening consultation. Interpretation services were available. During the screening consultation, the NP took a relevant family and personal history and determined eligibility for average and high-risk screening programs as per Ontario Health Cancer Care Ontario criteria (Cancer Care Ontario, 2025). The NP superscreener ordered all appropriate screening, including fecal immunohistochemical tests (FIT) for average risk colon cancer, mammograms for average risk breast, and pap tests for cervical screening, which were performed at episodic pap clinics delivered across the region. If high-risk criteria were satisfied, individuals were referred for colonoscopy instead of FIT, the High-Risk Ontario Breast Screening Program (OBSP), or the Ontario Lung Screening Program (OLSP). Individuals referred to High-Risk OBSP had mammograms ordered while they awaited assessment. Individuals already within High-Risk OBSP or OLSP who could not continue due to loss of their PCP were identified by these programs and submitted to the NP superscreener for re-referral to these programs. These same individuals were also offered screening consultations to assess for other unmet screening needs. The NP superscreener referred positive FIT results to colonoscopy for unattached individuals who had autonomously ordered these tests through Telehealth Ontario. The NP superscreener followed up on all results and referred on for further care when required.

### Data collection

This study was deemed quality improvement by the Ottawa Health Science Network Research Ethics Board as per the Tri-Council Policy Statement 2, Article 2.5; and therefore, ethics approval was not required. The NP superscreener collected data on the number of self-referrals, screening consultations, ordered and completed screens, screen results, and diagnosed cancers from July 2023 to July 2024. Abnormal results were considered to be screening tests requiring further follow-up: paps with atypical squamous cells of uncertain significance (ASCUS), low-grade squamous intraepithelial lesion (LSIL), or high-grade intraepithelial lesion (HSIL); mammograms which were Breast Imaging and Reporting System (BI-RADS) 0; positive FIT tests or colonoscopies with polyps or cancers; and Lung Imaging and Reporting System (Lung-RADS) 0 or ≥ 3. Proportions of participants screened in average and high-risk programs were compared to regional and provincial programs, as were positive predictive values (Ontario Health, 2024). Chi-squared tests with a significance level of 0.05 were used to determine differences.

### Participant demographics

Median total income, immigration status, and visible minority data were determined from the 2016 Census using the forward sortation area (FSA) of CSO participants (Government of Canada, 2021 2024). Income was expressed above or below that of Ottawa’s median household income before tax of $102,000 (Government of Canada, 2021). Primary care attachment rates for FSAs were determined (INSPIRE-PHC. 2023) Based on self-report, individuals were identified as “neverscreeners” if they had no prior cancer screening and overdue if they had not been screened as per the recommended interval. Ontario Marginalization Index (ON-Marg) information (2016) was determined for neverscreeners based on aggregate dissemination area, and the difference between first ON-Marg quintiles’ proportions compared to other quintiles was determined with chi-squared testing (Matheson et al.,  2023).

## Results

There were 871 referrals to CSO in its first year, 535 of which were eligible for the program (Table 1). In total, 527 screening consults were completed, with 786 screening tests ordered or arranged (257, 226, 222, and 81 for cervical, breast, colon, and lung cancers, respectively). Four individuals declined CSO services when contacted to book, and 12 screening tests were declined. Within the first year, 202 pap tests, 183 breast, 167 colon, and 42 lung screens were completed.

**Table 1:** Summary of screening tests ordered, screening status of participants and abnormal results

	Cancer screening program						
	Cervical	Breast		Colon		Lung	Total
		Average risk	High risk	Average risk	High risk		
**Screening tests ordered/performed**							
Screening offered/referred	257	135	50	107	76	43	668
Already enrolled no PCP, re-referred			41			38	79
Self-order FIT positive, no PCP					39		39
Screens completed within a year	202	183		57	110	42	594
**Abnormal results**							
	10	24		1	46	7	88
**Screening status**							
On schedule	32	104		105		23	264
Overdue	181	101		86		17	385
Never screened	44	73		53		49	219

**Fig. 1:**
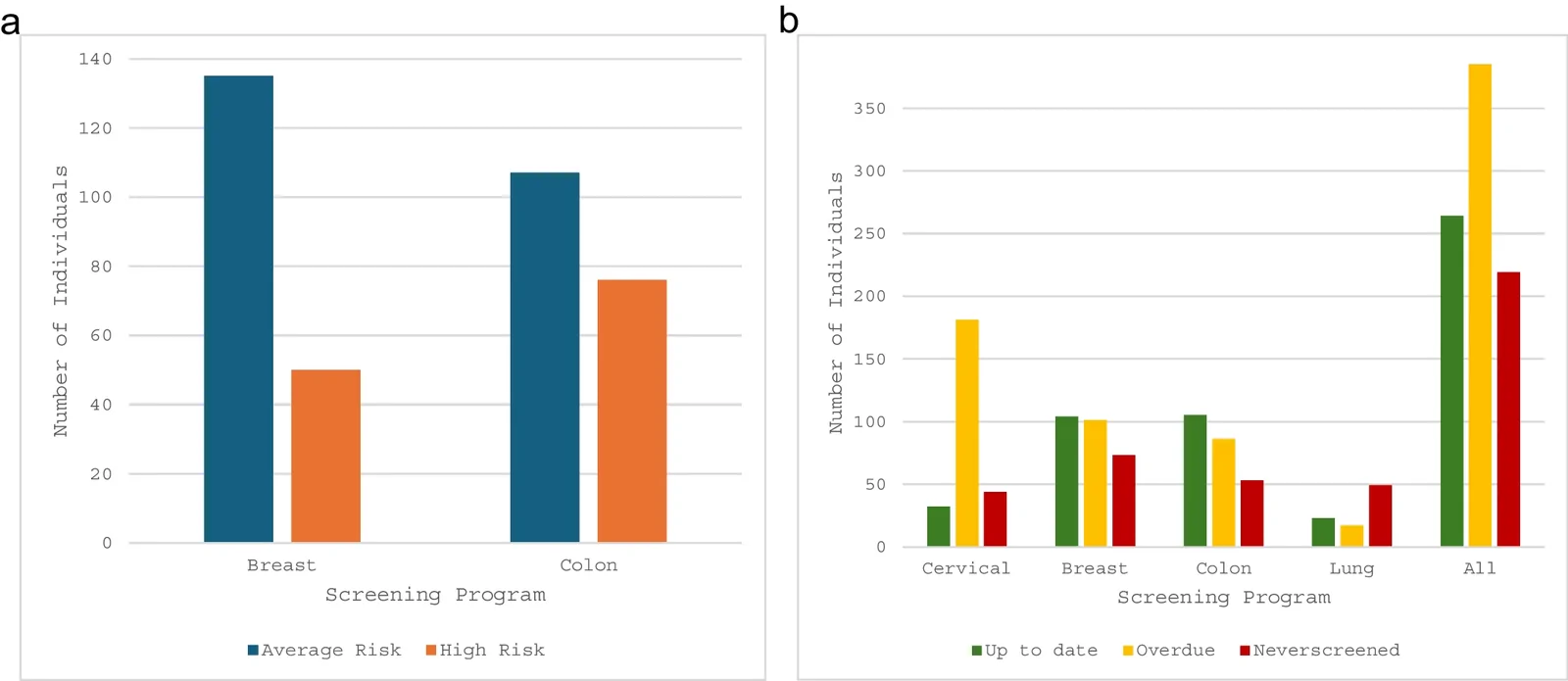
**a** Number of Champlain Screening Outreach participants referred to average risk and high-risk breast and colon screening programs. **b** Screening status for Champlain Screening Outreach participants

Fifty individuals were identified as eligible for high-risk OBSP, representing 27.3% of participants accessing breast screening (Table 1, Fig. 1a). One hundred and seven FIT tests were ordered, 57 of which were returned within the year. Seventy-six people, or 41.5% of those assessed for CRC screening, were identified as eligible for colonoscopy, with 71 completing the procedure within the year. There were 39 unattached individuals with positive autonomously ordered FITs, which the NP superscreener referred for colonoscopy. Finally, individuals who had lost their PCP were re-referred so that they could remain in the OLSP (38) and High-Risk OBSP (41). No centralized program exists for high-risk CRC, but individuals also self-referred to CSO to access colonoscopy after losing their PCP.

Screening status for all eligible cancer screening was documented during each consult (Table 1, Fig. 1b). One hundred and ninety-five (37.0%) of consults were neverscreeners. By individual screening program, 44 (17.1%), 73 (26.3%), 53 (21.7%), and 49 (55.1%) individuals were neverscreeners for cervical, breast, colon, and lung cancers, respectively. Eighty-one (70.4%), 101 (36.3%), 86 (35.2%), and 17 (19.1%) individuals were overdue for cervical, breast, colon, and lung cancer screening, respectively.

Ten individuals (5.0%) had abnormal cervical screening results, with low-grade cytology (ASCUS and LSIL) in six cases and four cases with clinical abnormalities (Table 1). Twenty-four individuals (13.1%) had abnormal mammogram results including two BI-RADS 3 and three BI-RADS 4. Forty-two colonoscopies (38.1%) were positive for polyps, with four cases of CRC diagnosed (10.3%) in self-ordered unattached FIT positive individuals. Seven individuals (16.7%) had Lung-RADS 0 or ≥ 3, four (9.5%) with Lung-RADS 0, one (2.4%) with Lung-RADS 3, and two (4.8%) were referred to the Diagnostic Assessment Program with 4b findings.

### Comparison with regional and provincial screening outcomes

The proportion of individuals determined to be eligible for high-risk CRC and breast screening compared to the screened population was significantly higher for CSO compared to Ontario (41.5% vs. 2.6%, *p *< 0.01 and 27.3% vs 1.1%, *p* < 0.01, respectively). Low-grade cytology rates were significantly higher in CSO compared to Ontario (3.06% vs 1.33%, *p* < 0.01). CSO had a significantly higher mammogram recall rate of 13.1% compared to 9.2% (*p *< 0.01) for Ontario and 8.9% (*p* = 0.01) for Champlain region. The FIT return rate of 53.3% was less than the provincial rate of 65.1% for 2021/2022 (Cancer Care Ontario, personal communication), and the FIT abnormal rate for CSO was significantly lower (1.8%) than observed for Champlain (4.3%, *p* < 0.01). The rate of diagnosis of CRC in positive FITs was higher in CSO (10.3%), compared to Ontario (5.3%) and Champlain (6.3%).

### Participant demographics

**Fig. 2:**
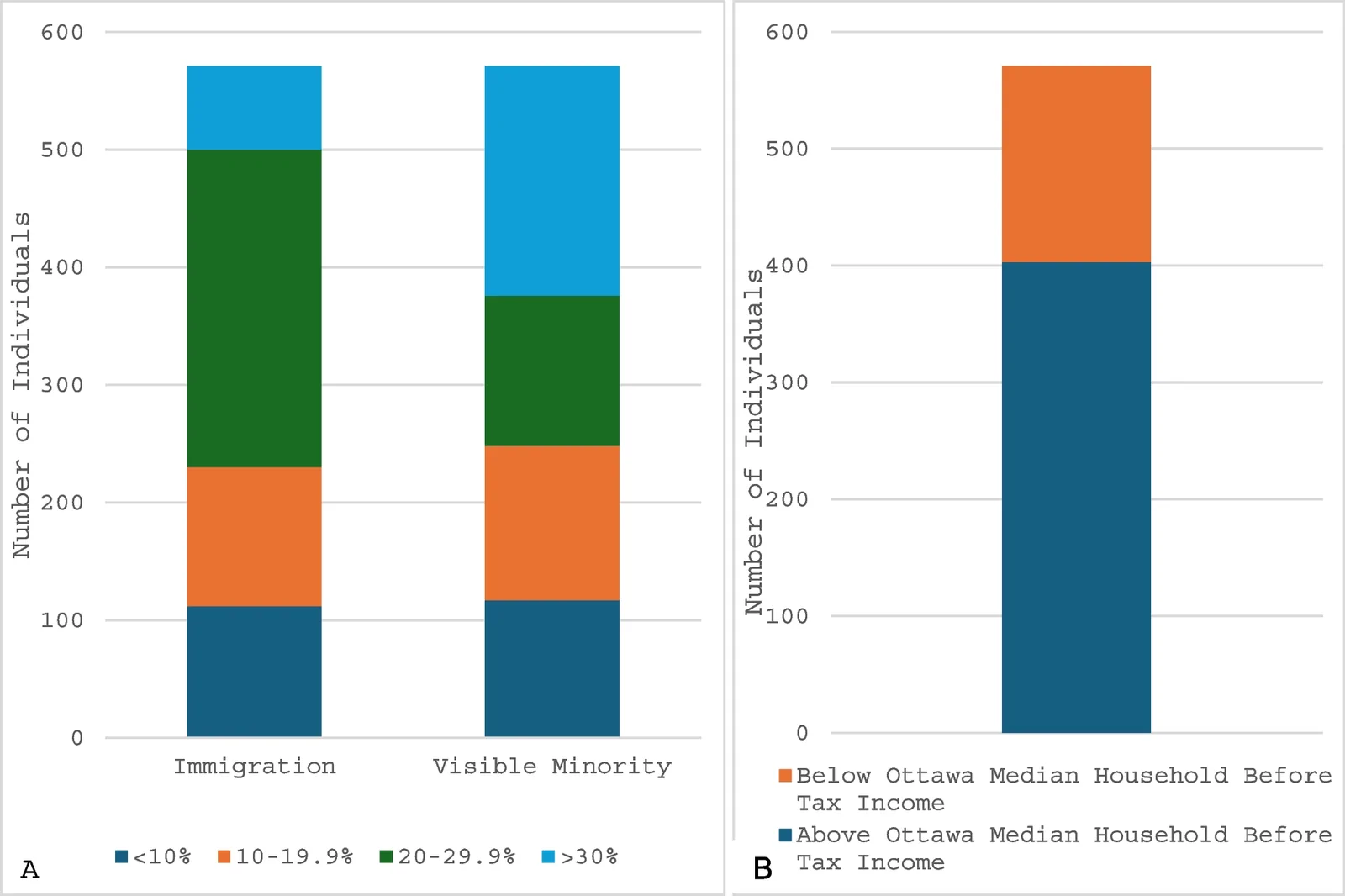
**a** Number of Champlain Screening Outreach participants according to percentage of neighborhood immigration and visible minority prevalence as determined by participant forward sortation area. **b** Number of Champlain Screening Outreach participants according to median household before tax income in comparison to the Ottawa median, as determined by forwards sortation area

The largest proportion (34.1%) of CSO participants resided in neighborhoods characterized by a greater than 30% visible minority prevalence, and 47.3% of participants lived in areas with an immigration rate of 20 to 30% (Fig. 2a and b). The majority (70.6%) of CSO participants resided in neighborhoods with a lower median household before tax income than that of Ottawa. Furthermore, 26.3% of CSO participants lived in neighborhoods with the highest quintile for uncertain attachment to a PCP (19.6–26.3% unattached) and about half (49.0%) lived in the highest two quintiles for unattachment (> 16.7% unattached).

**Fig. 3:**
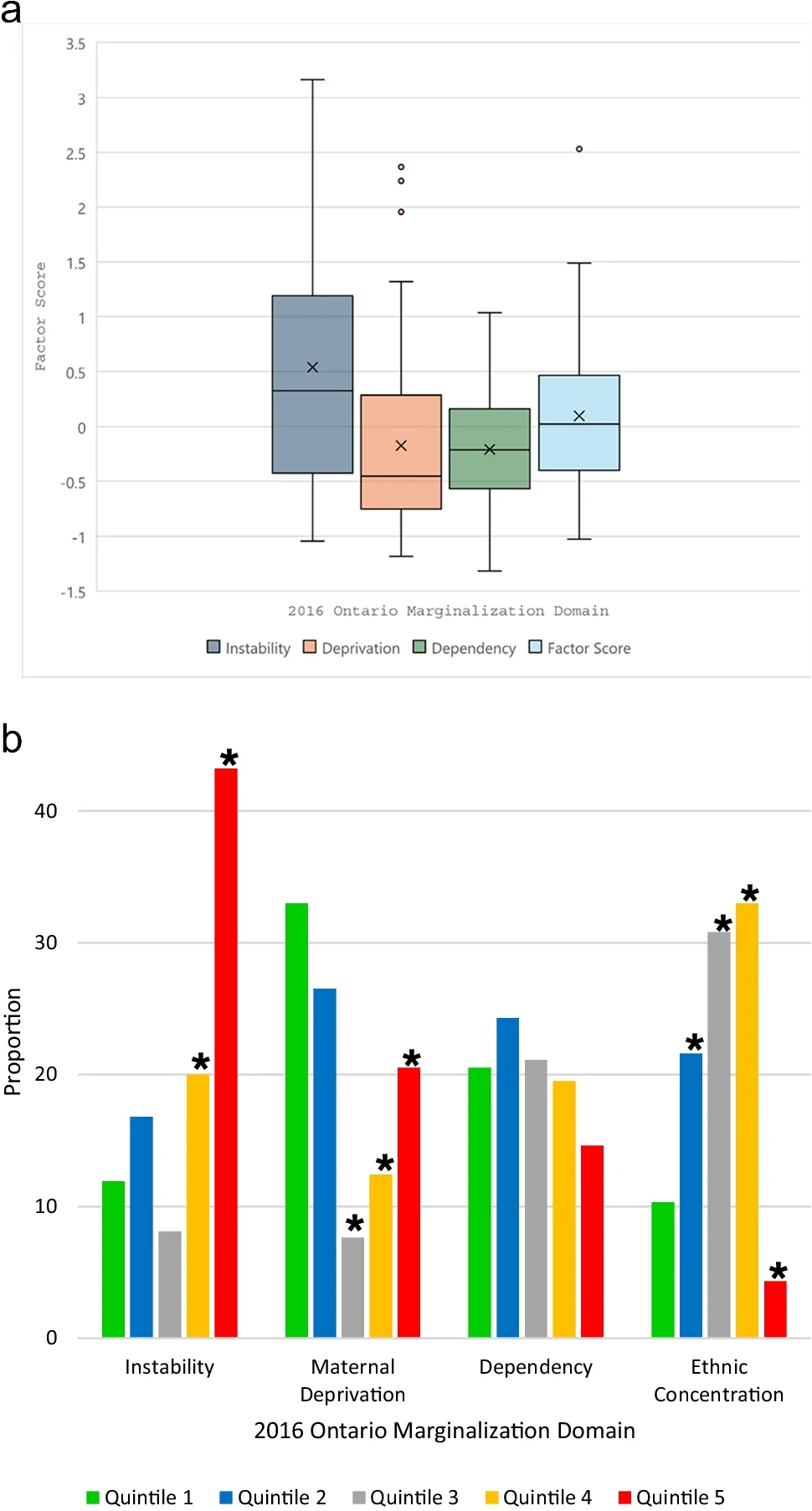
**a** Box plot of 2016 Ontario Marginalization factor scores for individuals identifying as neverscreeners in the Champlain Screening Outreach program. Higher factor scores denote higher marginalization. **b** Proportions of Champlain Screening Outreach neverscreeners by 2016 Ontario Marginalization quintiles. Stars signify quintiles where proportion is significantly different than in quintile one

Mean 2016 ON-Marg factor scores for neverscreener individuals with standard error of mean were as follows: instability (0.54 ± 0.08), material deprivation (−0.17 ± 0.06), dependency (−0.21 ± 0.4), and ethnic concentration (0.01 ± 0.05) (Fig. 3a). There was no significant difference in proportion by quintile for dependency (*p* > 0.05 for all) (Fig. 3b). There were significantly more individuals in quintile 1 than in quintiles 3 (*p* < 0.01), 4 (*p* < 0.01), and 5 (*p* = 0.01) for material deprivation. The proportion of individuals in quintiles 2, 3, and 4 for ethnic concentration was significantly higher than quintile 1 (*p* < 0.01 for all); however, the proportion of individuals in the 5^th^ quintile was significantly lower than the 1^st^ quintile (*p* = 0.05). The proportions of individuals in the 4^th^ and 5^th^ quintiles for instability were significantly higher than in quintile 1 (*p* = 0.05 and *p* < 0.01, respectively).

### Cost

The total cost for CSO for the first year was $118,080, of which $8,934 was one-time capital start-up costs. Expenses were as follows: $107,811.53 for salary including benefits; $1335 for travel and $1335 for medical supplies. Capital costs were $1623 for printing, $1647 for translation, and $4289 for computer expenses. This yielded a cost per consult of $207.10, or a cost per screening test of $138.86.

## Discussion

In its inaugural year, the CSO program successfully increased the accessibility of cancer screening for unattached individuals in the Champlain region. The program effectively identified populations underserved by conventional healthcare practices, with over one-third of the individuals identifying as neverscreeners. CSO underscored how the primary care crisis has prevented already identified high-risk patients from continuing with appropriate screening, and how improved risk identification results in increased referral rates to high-risk cancer screening programs. Individuals screened through CSO had higher rates of abnormal screen results than provincial and regional rates. The CSO data provided a valuable means to explore the sociodemographic context of unattached and neverscreener populations.

The higher rate of abnormal screen results seen in cervical, breast, and colon CSO individuals compared to provincial rates likely reflects that three-quarters of the CSO population were never/underscreened. Rates of positive cancer screens are higher in underscreened populations (Bernard et al., 2015), and communities facing systemic barriers to accessing preventive healthcare are reflected in disparities in cancer outcomes (Scott & Hoskin, 2024). Abnormal results could also be related to optimized identification of individuals for high-risk screening activities. The higher mammogram recall rate in CSO may be related to higher recall rates for initial screens compared to subsequent tests (Ontario Health, 2024). Given that Champlain regional recall rates are lower than the provincial average, the observed CSO rates are unlikely related to regional quality issues in mammographic reporting. The FIT return rate of 53.3% appears low given that individuals had self-referred and undergone a screening consultation; however, FIT return rates observed in underserved communities are documented around 40% (Coronado et al., 2018). Programmatic reminders for screening completion are effective in underserviced populations and could be considered a future CSO initiative to enhance FIT return rates, along with onsite distribution of FIT kits (Murphy et al., 2022; Nanda et al., 2022; O’Leary et al., 2023).

The cost per screen does not reflect the full value of the CSO program, as in this pilot, the NP superscreener was not initially fully booked, and we did not capture cost savings due to earlier stage screen-detected cancers. Breast cancer screening has been shown to save health systems money due to the lesser treatment costs noted with earlier stage screen-detected cancers (Wilkinson et al., 2025). With greater participation over time, it will be important to understand the net cost of a screening outreach program given the stage-shift associated with screening and associated lower cancer treatment costs.

Over half of individuals screened for lung cancer were neverscreeners, highlighting the ability of CSO to find and identify high-risk individuals effectively. Appropriate identification of individuals for high-risk screening programs involves both an awareness of these programs and a thorough personal and family history. The transition from annual physical to periodic health examination practically results in fewer occasions for preventative health discussions. Cancer screening dialogues with PCPs instead tend to occur opportunistically at acute medical appointments, without dedicated time for episodic reviews of family history and evolving risk factors. Indeed, in one study, patients overdue for cervical screening had an average of four clinic visits with primary care in the preceding months, but no pap tests performed (Ogunwale et al., 2016). The increased referrals to high-risk programs through CSO demonstrate that dedicated screening consultations, performed by an NP with up-to-date knowledge of screening programs and criteria, more effectively identify individuals than ad hoc PCP activities, while simultaneously conserving valuable family physician access.

CSO participant information can be used to comprehend the demographics of unattached individuals. These patients lived in neighborhoods characterized by high rates of visible minority and immigration status and low incomes, as noted in previous studies of unattached individuals (Matthews et al., 2024; Marshall et al., 2022). Interestingly, the profile of neverscreeners in CSO is different from the overall unattached population. Economic factors were lef neighborhood and supportsss important for neverscreeners, with the majority (60%) within the two most favorable quintiles of material deprivation. Only 4% of neverscreeners were in the highest quintile for ethnic concentration. Instability appeared to be the most important factor for neverscreeners, with 63.2% within the 4^th^ and 5^th^ quintiles. Instability refers to high rates of family or housing instability and is related to the quality of neighborhood and supports (Matheson, 2018). Individuals from high-instability areas are more likely to face challenges such as frequent changes in residence, lack of social supports, and difficulty maintaining consistent access to healthcare services (Kushel et al., 2006). These factors can correlate with poorer engagement in preventive care, given the need to deprioritize health needs or overcome logistical barriers. Traditional practices of mailing screening reminders may not be effective for populations with high levels of instability. The insights gained about neverscreeners will be crucial in guiding future outreach efforts, allowing for more effective targeting of vulnerable and high-yield populations.

Screening programs with the ability to self-refer (breast, colon) had the lowest rates of overdue for screening. Alternatively, 87.5% of individuals accessing cervical screening through CSO were neverscreeners/overdue, highlighting difficulties for unattached individuals to access cancer screenings that are reliant on a clinician. The introduction of HPV self-screening could remove barriers to access in the setting of a primary care crisis and should be considered to improve health equity. However, the finding of four colon cancers in patients who autonomously requested FIT kits emphasizes the importance of a centralized process with an embedded clinician to manage abnormal results and liaise with patients.

Although initial results from the CSO are encouraging, awareness of the program was building in its inaugural year, and only a part-time NP was available for screening consults. As such, the small sample size constrains the generalization of these findings when comparing to provincial and regional benchmarks. Demographic data was analyzed based on geography and may not precisely represent individual neverscreeners. To minimize this limitation, the smallest possible spatial units were used. Long-term outcomes, including regional screening rates, cancer incidence, and survival rates will be important to monitor, and measuring patient satisfaction and cost-effectiveness could also provide valuable insights for scaling the program to other regions.

CSO is an innovative approach which increases access for unattached individuals and prioritizes equity for cancer screening, while optimizing resource use. The program shows the benefits of collaboration between the cancer screening system and public health organizations. CSO had a superior ability to access never-/underscreened individuals, and identify people eligible for high-risk cancer screening. CSO successfully reduced barriers to cancer screening by offering virtual services and a “one-stop” shop for all provincial screening programs, at the same time generating valuable information about the populations it served. CSO provides a centralized means to manage self-ordered test results, which will become critical in a transition to HPV self-screening. As the primary care crisis continues to deepen, centralized, proactive screening programs such as CSO could be scaled nationally. Such programs should play an important role in primary care teams, conserving scarce family physicians’ time while simultaneously improving early detection and cancer outcomes and advancing health equity.

## Implications for policy and practice

What are the innovations in this policy and program?

- (why 2022?) CSO moves cancer screening outside of a PCP-centered model, ensuring continued access during a primary care crisis
- Collaboration with public health units and community partners with local access to cervical screening increases education and uptake in typically underscreened populations
- Improved identification of high-risk screening populations
- Enhanced accessibility with self-scheduling and phone consults
- Centralised management of abnormal screen results
- Improved understanding of neverscreened populations

What are the burning research questions for this innovation?

- Could this screening approach offer a provincial or national solution to the primary care crisis?
- Will CSO result in increased screening rates within our region?
- Will this program improve cancer outcomes such as increased earlier stage diagnosis and better survival?
- Do never-/underscreeners screened through CSO show long-term screening adherence?
- Is CSO a cost-effective means to deliver cancer screening?
- Could the CSO model be expanded to provide other services, such as HPV vaccination?
- What methods could be implemented to improve the FIT kit return rate?
- Could HPV self-screening be integrated into the CSO model to improve cervical screening rates?

## Data Availability

Data available on request.

## Code availability

n/a.
